# Whipple's Disease Affecting Ileal Peyer's Patches: The First Case Report

**DOI:** 10.1155/2019/1509745

**Published:** 2019-06-23

**Authors:** Sabah Sid'Amar, Giacomo Puppa

**Affiliations:** ^1^Department of Clinical Pathology, Promed SA, Medical Laboratory, Marly/Fribourg, Switzerland; ^2^Department of Clinical Pathology, Geneva University Hospital, Geneva, Switzerland

## Abstract

Whipple's disease is a rare chronic systemic bacterial infectious disease which can affect multiple organs, with a wide clinical spectrum encompassing many symptoms presenting in various forms and combinations. In the cases where the gastrointestinal tract is implicated, the more frequent localizations involve the small bowel, especially the duodenum. A case of a 67-year-old man who underwent clinical investigation after presenting with a progressive weight loss and showing a hypercapting right paracoeliac adenopathy at PET-CT scan is reported herein. A gastroscopy and a colonoscopy were done. The biopsies of the endoscopically normal ileal mucosa encompassed some submucosal Peyer's patches. Histological examination of this lymphoid tissue revealed several foamy macrophages which turned out positive on periodic acid-Schiff special staining. Polymerase chain reaction of the microdissected lymph follicles allowed for confirming Whipple's disease diagnosis. A targeted antibiotic treatment administrated to the patient led to a rapid clinical improvement. This finding of a previously unreported localization of infected macrophages in Whipple's disease suggests that sampling the organized mucosal-submucosal lymphoid tissue may increase the diagnostic yield in endoscopic biopsies.

## 1. Background

Whipple's disease (WD) is a rare chronic systemic bacterial infectious disease caused by* Tropheryma whipplei*. Its diagnosis is rendered difficult because of several reasons and often is established only after many years.

Symptoms are not specific as many organs can be affected, both gastrointestinal and extragastrointestinal [1–3].

Endoscopic findings are also heterogeneous ranging from normal to more typical yellowish plaques or white spots [4, 5].

Gastrointestinal localizations include more frequently the small bowel, particularly the duodenum. Random biopsies taken from the duodenum and the jejunum may show pink-coloured, foamy macrophages filling the lamina propria with expansion and distortion of the villi.

Periodic acid-Schiff (PAS) positive macrophages outside the intestinal mucosa can be found in enlarged mesenteric or extragastrointestinal lymph nodes [2, 6].

We present in this case a unique, unreported WD localization in Peyer's patches.

## 2. Case History

A 67-year-old man underwent clinical investigation after presenting with a progressive weight loss of 13 kg across 6 years without any other symptoms. Clinically, there were no relevant gastrointestinal manifestations. The patient showed no manifestation of any articular pain or neurological deficiency. A PET-CT scan highlighted a hypercapting right paracoeliac adenopathy. The patient underwent a gastroscopy and a colonoscopy primarily to exclude a lymphoma versus an inflammatory process.

Endoscopic investigations of the upper and lower gastrointestinal tract showed two small (approximately 5 mm) polyps on the gastric fundus and on the right colon. Histological examination of the two polyps identified a fundic gland polyp and a low-grade dysplastic tubular adenoma, respectively.

The duodenal tract showed no endoscopic anomaly and four random biopsies were done, sampling the mucosa and a small amount of the submucosa, which were histologically totally normal.

**Figure 1:**
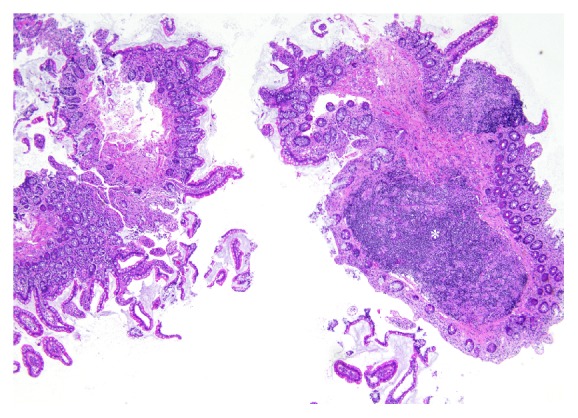
Biopsies of the ileal mucosa appearing normal structurally and cellularly. The submucosa is occupied by a Peyer's patch (asterisk). Hematoxylin and eosin original magnification, 40x.

Biopsies of the endoscopically normal ileal mucosa showed a structurally and cellularly normal mucosa. The submucosa was also sampled, encompassing few Peyer's patches (Figure 1).

**Figure 2:**
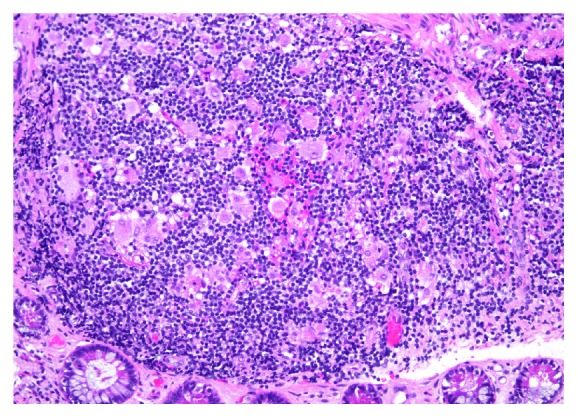
Peyer's patch at higher magnification (hematoxylin and eosin 200x) contains several scattered pink-coloured, foamy macrophages.

At higher magnification, several pink foamy macrophages were observed in the lymph follicles (Figure 2).

**Figure 3:**
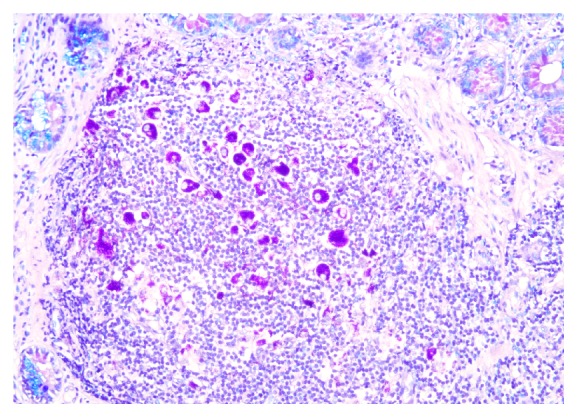
A periodic acid-Schiff (PAS, 200x) special stain displays the PAS-positivity of the macrophages.

The PAS staining, performed routinely on intestinal biopsies, highlighted the characteristically pink-coloured macrophages (Figure 3).

**Figure 4:**
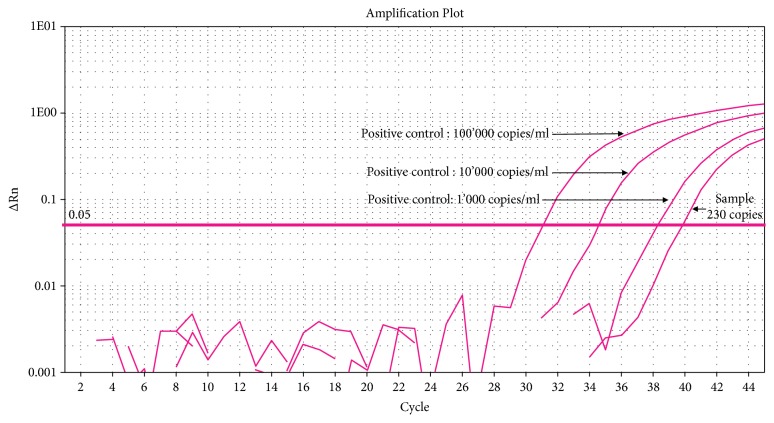
Amplification plot from the ileal sample concerned during the real-time PCR showing the amplification curve of specific* T. whipplei* DNA to 230 copies/ml.

A real-time quantitative polymerase chain reaction (qPCR) was performed on the microdissected lymph follicles, resulting in 230 copies/ml of* Tropheryma whipplei* DNA (Figure 4). Diagnosis of WD was therefore established.

A targeted antibiotic treatment plan was applied, which included parenteral administration of Ceftriaxone (2 g) once a day for one month, followed by oral maintenance with 1 tablet of Bactrim forte twice a day for one year. The patient was able to gain 2 kg of body weight after one month of therapy.

## 3. Discussion

Classic WD is a rare systemic chronic infectious disease caused by the bacterium* Tropheryma whipplei*, an intracellular Gram-positive bacillus of the Actinomycetes family [7].

The disease affects mostly middle-aged men with a greater prevalence in Caucasian populations. The estimated annual incidence is known to be low (1:1,000,000) [4, 8], considering this bacterium being well known to be ubiquitous in the environment and common in humans [1, 7, 9] as a commensal bacterium [10]. It should be taken therefore into account regularly as its progression may lead to a fatal outcome if left untreated [4, 11].

The transmission pathway of this bacterium takes place among humans by oro-oral and orofaecal routes, linked to hygienic habits and rural environments [9–11]. It is intriguing that healthy individuals may carry* Tropheryma whipplei* without necessarily ever developing the disease.

The host-specific dysfunction of the intestinal macrophages could contribute towards a chronic infection [4] in the way that these macrophages are unable to degrade the bacterial antigens efficiently after the phagocytosis, which does not seem to be impaired [4, 12, 13].

The classic clinical manifestations present as a trio of symptoms, including an initially chronic arthropathy (arthralgia, arthritis), weight loss, and diarrhoea/malabsorption syndrome. Several organs can be affected, mainly the heart, central nervous system, lung, and lymph nodes [2, 4]. Gastrointestinal localizations include more frequently the small bowel, but also the stomach, oesophagus, and colon [3, 14].

Many symptoms present in various forms and combinations. Not infrequently, cases present with localized forms (endocarditis, encephalitis) without gastrointestinal symptoms [2, 7, 9, 13, 15–17]. Diagnosis of WD, therefore, remains challenging despite the recent advancements in medical and technical tools [15, 16, 18].

Another complication is due to the fact that there might be a long time span from the presentation of the first symptoms to the full-blown clinical manifestation of the disease [4, 11, 16], with an average latency of 6-8 years [4].

Endoscopically, the intestinal mucosa may show erosions and diffuse white yellowish shaggy patches or may appear as normal [4, 5].

Histological diagnosis relies on identifying pink-coloured, foamy macrophages filling the lamina propria displaying an intense positivity on PAS special staining. However, as PAS-positive macrophages in the GI tract are not pathognomonic of WD, it is necessary to rule out other diagnoses and to rely on further ancillary stains and additional techniques, to get to the correct diagnosis.

The differential diagnosis includes predominantly infection by Mycobacterium avium Complex, wherein the macrophages turn out positive on PAS staining and Ziehl-Neelsen staining, the latter being negative in WD [19]. Other very rare infections may show PAS-positive macrophages, as* Rhodococcus equi* which is also Gram-positive,* Bacillus cereus*,* Corynebacterium*, Histoplasmosis, or even fungi, as well as Malakoplakia, but also appearing with other histological features [4, 12].

Further additional techniques for diagnosis are* T. whipplei*-specific immunohistochemistry,* T. whipplei*-specific PCR, and electron microscopy [17, 19], the former two being a gold standard choice of diagnostic tools because of their high sensitivity and specificity in detecting* T. whipplei* [9, 12, 16].

In this case report, the nonspecific clinical picture of a long-standing state with progressive weight loss and clinical investigations showing a hypercapting paracoeliac lymph node led to a gastroscopy and a colonoscopy. The PAS-positive macrophages found in Peyer's patches from the end ileum permitted the diagnosis of WD which was confirmed by specific PCR.

The PAS-positive macrophages generally are encountered in the lamina propria of the small bowel mucosa, most often in the duodenum and sometimes in the jejunum [17], with rare cases reported in the ileum [5, 16, 17]. In this case, intestinal random biopsies from a normal endoscopic mucosa were taken, demonstrating a histologically normal duodenum and revealing the macrophages in the underlying ileal submucosa in the lymph follicles.

During the clinical work-up, a paracoeliac lymphadenopathy was found on the radiological images, whose nature of its contents can be questionable. Finally, no biopsy of this lymphadenopathy has been performed, since the diagnosis has been established yet on the ileal biopsies. We can still wonder if it was contaminated by* T. whipplei *and even raise the matter of the possibility of a primary lymph node involvement by WD, the reason why an abdominal lymphadenopathy could motivate endoscopic investigations with performing intestinal biopsies for diagnostic purposes [6]. Involvement of the abdominal lymph nodes by WD is not uncommon by far. However, peripheral lymph nodes involved by WD as a sole clinical manifestation are rare [19]. The importance of analysing a mesenteric lymph node by specific PCR to detect* T. whipplei *in the absence of suggestive PAS-positive macrophages on histological analysis has been described [17].

However, despite the well-known lymphoid tissue trophicity and the key immunologic role of Peyer's patches against gut antigens and bacteria [20], we remain puzzled that, to the best of our knowledge, WD has never been reported as localized in Peyer's patches. The correct diagnosis permitted an appropriate treatment for the patient, with rapid clinical improvement.

## 4. Conclusion

We report here the first case of WD with contaminated macrophages found in ileal Peyer's patches. The morphological finding of foamy macrophages displaying a positivity on the ancillary PAS staining led to the suspicion of the diagnosis of WD, which was confirmed by PCR. Despite literature suggesting the duodenum as the most frequent area of localization, the normal ileum, including the associated lymph follicles, should also be sampled to shorten the diagnostic latency; otherwise, this potentially lethal outcome disease could remain unrecognized. Additionally, we wonder if it would be pertinent to target the nodular mucosa (which normally encompasses Peyer's patches) from the ileum for diagnostic purposes. An indication to biopsy the small intestine could be a clinical presentation with an abdominal lymphadenopathy.
